# Development and external validation of automated detection, classification, and localization of ankle fractures: inside the black box of a convolutional neural network (CNN)

**DOI:** 10.1007/s00068-022-02136-1

**Published:** 2022-11-14

**Authors:** Jasper Prijs, Zhibin Liao, Minh-Son To, Johan Verjans, Paul C. Jutte, Vincent Stirler, Jakub Olczak, Max Gordon, Daniel Guss, Christopher W. DiGiovanni, Ruurd L. Jaarsma, Frank F. A. IJpma, Job N. Doornberg, the Machine Learning Consortium, Kaan Aksakal, Britt Barvelink, Benn Beuker, Anne Eva Bultra, Luisa e Carmo Oliviera, Joost Colaris, Huub de Klerk, Andrew Duckworth, Kaj ten Duis, Eelco Fennema, Jorrit Harbers, Ran Hendrickx, Merilyn Heng, Sanne Hoeksema, Mike Hogervorst, Bhavin Jadav, Julie Jiang, Aditya Karhade, Gino Kerkhoffs, Joost Kuipers, Charlotte Laane, David Langerhuizen, Bart Lubberts, Wouter Mallee, Haras Mhmud, Mostafa El Moumni, Patrick Nieboer, Koen Oude Nijhuis, Peter van Ooijen, Jacobien Oosterhoff, Jai Rawat, David Ring, Sanne Schilstra, Jospeph Schwab, Sheila Sprague, Sjoerd Stufkens, Elvira Tijdens, Michel van der Bekerom, Puck van der Vet, Jean- Paul de Vries, Klaus Wendt, Matthieu Wijffels, David Worsley

**Affiliations:** 1 Department of Orthopaedic Surgery, Groningen University Medical Centre, Groningen, The Netherlands; 2 Department of Surgery, Groningen University Medical Centre, Groningen, The Netherlands; 3 Department of Orthopaedic & Trauma Surgery, Flinders Medical Centre, Flinders University, Adelaide, Australia; 4 Australian Institute for Machine Learning, Adelaide, Australia; 5 College of Medicine and Public Health, Flinders University, Adelaide, Australia; 6 Department of Neurosurgery, Flinders Medical Center, Adelaide, Australia; 7 Institute of Clinical Sciences, Danderyd University Hospital, Karolinska Institute, Solna, Sweden; 8 Massachusetts General Hospital, Boston, USA; 9 Harvard Medical School, Boston, USA

**Keywords:** Artificial Intelligence, CNN, Ankle, Lateral Malleolus

## Abstract

**Purpose:**

Convolutional neural networks (CNNs) are increasingly being developed for automated fracture detection in orthopaedic trauma surgery. Studies to date, however, are limited to providing classification based on the entire image—and only produce heatmaps for approximate fracture localization instead of delineating exact fracture morphology. Therefore, we aimed to answer (1) what is the performance of a CNN that detects, classifies, localizes, and segments an ankle fracture, and (2) would this be externally valid?

**Methods:**

The training set included 326 isolated fibula fractures and 423 non-fracture radiographs. The Detectron2 implementation of the Mask R-CNN was trained with labelled and annotated radiographs. The internal validation (or ‘test set’) and external validation sets consisted of 300 and 334 radiographs, respectively. Consensus agreement between three experienced fellowship-trained trauma surgeons was defined as the ground truth label. Diagnostic accuracy and area under the receiver operator characteristic curve (AUC) were used to assess classification performance. The Intersection over Union (IoU) was used to quantify accuracy of the segmentation predictions by the CNN, where a value of 0.5 is generally considered an adequate segmentation.

**Results:**

The final CNN was able to classify fibula fractures according to four classes (Danis-Weber A, B, C and No Fracture) with AUC values ranging from 0.93 to 0.99. Diagnostic accuracy was 89% on the test set with average sensitivity of 89% and specificity of 96%. External validity was 89–90% accurate on a set of radiographs from a different hospital. Accuracies/AUCs observed were 100/0.99 for the ‘No Fracture’ class, 92/0.99 for ‘Weber B’, 88/0.93 for ‘Weber C’, and 76/0.97 for ‘Weber A’. For the fracture bounding box prediction by the CNN, a mean IoU of 0.65 (SD ± 0.16) was observed. The fracture segmentation predictions by the CNN resulted in a mean IoU of 0.47 (SD ± 0.17).

**Conclusions:**

This study presents a look into the ‘black box’ of CNNs and represents the first automated delineation (segmentation) of fracture lines on (ankle) radiographs. The AUC values presented in this paper indicate good discriminatory capability of the CNN and substantiate further study of CNNs in detecting and classifying ankle fractures.

**Level of evidence:**

II, Diagnostic imaging study.

## Introduction

Convolutional neural networks (CNNs) are increasingly being developed in orthopaedic trauma surgery for automated detection and classification of fractures [1–11]. General benefits include the fact that they (a) do not suffer from mental or physical fatigue compared to clinicians, (b) are consistent in their assessment because they are not limited by surgeon bias or poor inter-surgeon reliability [12–15], and (c) can perform at or above the level of consensus agreement from a panel of experienced surgeons and radiologists [1, 5, 10, 11, 16]. To date, most studies that have developed CNNs for fracture detection and classification primarily apply models that classify based on the entire or cropped input image [1, 5, 7, 10, 11, 16–18]. In contrast, newer computer vision techniques can detect, segment (i.e. exact delineate the suggested location of the fracture (Fig. 5)), and classify fracture patterns.

Automated delineation of fracture lines gives us insight into what the algorithm ‘sees’, and may help foster clarity for the as yet ill-defined role of artificial intelligence (AI) in the field of computer vision for fracture recognition [19, 20]. The next level of CNN studies in our field report detailed segmentation by a CNN of the second intact metacarpal [21] on plain radiographs, vertebrae on computed tomography (CT) [22], and femora on magnetic resonance imaging (MRI) [23, 24]. To the best of our knowledge, however, detailed segmentation of fracture lines on radiographs has yet to be reported.

In 2020, Olczak and colleagues successfully applied a CNN for ankle fracture classification [8] using the image-level classification model ResNet [25], but without automated delineation of the fracture. It remains the only fracture recognition paper for patients sustaining ankle trauma to date. Ideally, CNNs should combine object detection with segmentation, and thus offer localization and classification simultaneously—for example to better guide junior doctors during their early learning curves by presenting an exact visual outline of the fracture line itself. In addition, CNNs are often trained with large datasets without selecting cases that facilitate the most efficient training rate for the CNN (i.e. learning rate). This results in a large portion of unnecessarily labelled and/or annotated cases, because these contribute minimally to the performance of the model.

Therefore, we aimed to develop a CNN that detects (i.e. fracture yes/no), classifies (according to AO/OTA 44/Weber A, B and C [26]), and localizes (with exact delineation/segmentation of an ankle fracture). The following questions will be answered in this paper: (1) What are the diagnostic performance characteristics (accuracy, sensitivity, specificity) and area under the receiver operator characteristic curve (AUC) of a CNN that classifies, localizes, and segments a lateral malleolus ankle fracture?, (2) Is this CNN externally valid?, and (3) Does application of a preliminary CNN that selects an appropriate training set result in an efficient training rate for the CNN?

## Materials and methods

This study was approved by our Regional Review Board, according to the Declaration of Helsinki under number 13991.

### Guidelines

This study was conducted according to the Guidelines for Developing and Reporting Machine Learning Predictive Models in Biomedical Research [27] as well as the CONSORT-AI [28] the SPIRIT-AI [29], MI-CLAIM [30], and the CAIR checklist [31].

### Dataset

**Fig. 1:**
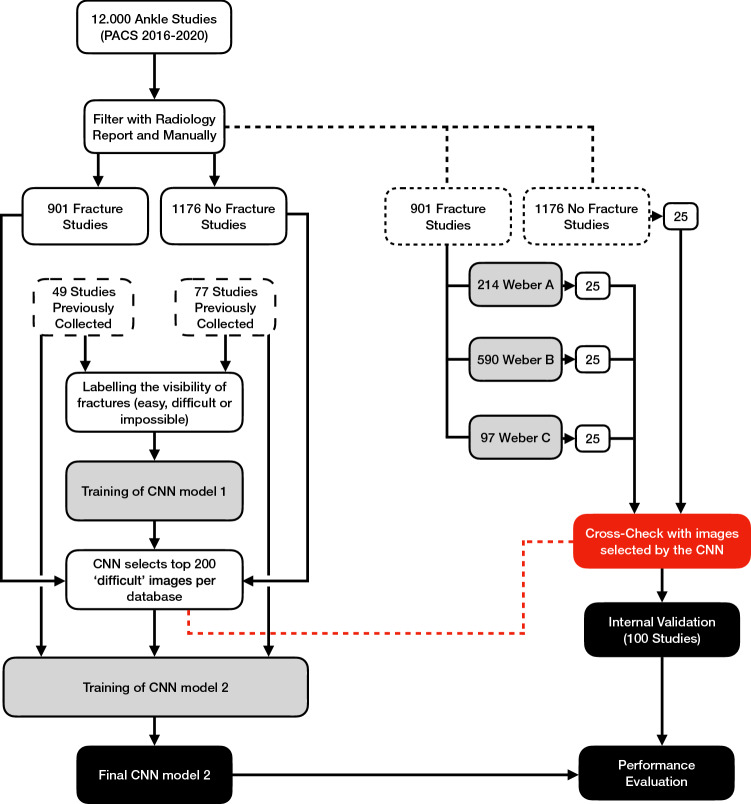
Workflow used to create the final convoluted neural network (CNN) for the classification of ankle fractures. This involves a two-stage approach. An initial CNN was trained to select cases that were considered difficult—for example, fractures that were hard to appreciate—for classification. Subsequently, the final CNN was trained using these radiographs selected by the former CNN

For this study, 12.000 radiographic ankle examinations with standard views (AP, Mortise and Lateral) were retrospectively collected from our Level 1 Trauma Centre, between January 2016 and December 2020. Studies were filtered using keywords in radiology reports to create an index database containing isolated fibular fractures and a non-fracture database (Fig. 1).

Three independent observers manually reviewed and classified the radiographs according to the AO/OTA 44/Weber A, B, and C [26], thereby excluding malleolar fractures where the tibia was involved. Any disagreements were resolved by discussion with a fourth independent senior observer. Data curation further excluded radiographs with fractures and pathology other than a fibular fracture, old fractures, presence of callous or cast, radiographs of poor quality (i.e. radiographs of patients that would be sent back to radiology in clinic), open physes, radiological views of insufficient quality, and presence of plates or screws.

### Preliminary CNN model

To improve efficiency in labelling and segmentation, a preliminary CNN was trained to provide model-assisted labelling and annotations. For the annotation task, the DeepLab V3 + [32] architecture with MobileNet V2 [33], pre-trained on ImageNet [34] data, was used. For the classification task, a separate CNN with a MobileNet V2 [33] backbone and a softmax classifier were used. Training data for the preliminary CNN consisted of 147 radiographs with a fracture and 228 without a fracture. Using Labelbox [35], the images were manually labelled for visibility of the fracture (easy, difficult, or impossible) and annotated by two independent observers for the following: shape of tibia/fibula and fracture. Bounding boxes were created around the borders of these respective annotations.

### Final CNN model (Fig. 2)

For final model development, the Detectron2 [36] implementation of the Mask R-CNN was used. The backbone of the Mask R-CNN model was set to the Microsoft Research Asia version ResNet-50 [37], pre-trained on ImageNet [34]. The ResNet-101 variation of the backbone was tested but did not result in significant improvement.

**Fig. 2:**
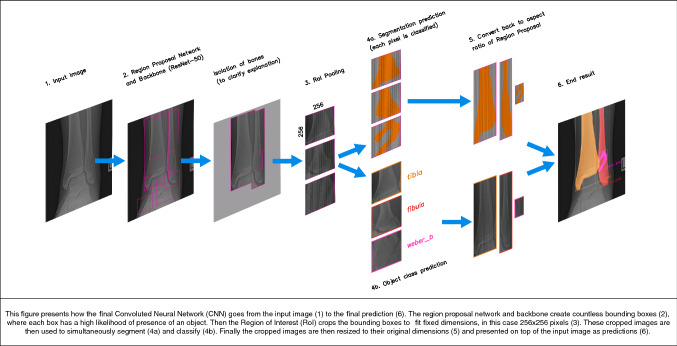
This figure presents how the final convoluted neural network (CNN) goes from the input image (1) to the final prediction (6). The region proposal network and backbone create countless bounding boxes (2), where each box has a high likelihood of the presence of an object. Then, the region of interest (RoI) crops the bounding boxes to fit fixed dimensions, in this case 256 × 256 pixels (3). These cropped images are then used to simultaneously segment (4a) and classify (4b). Finally, the cropped images are then resized to their original dimensions (5) and presented on top of the input image as predictions (6)

**Fig. 3:**
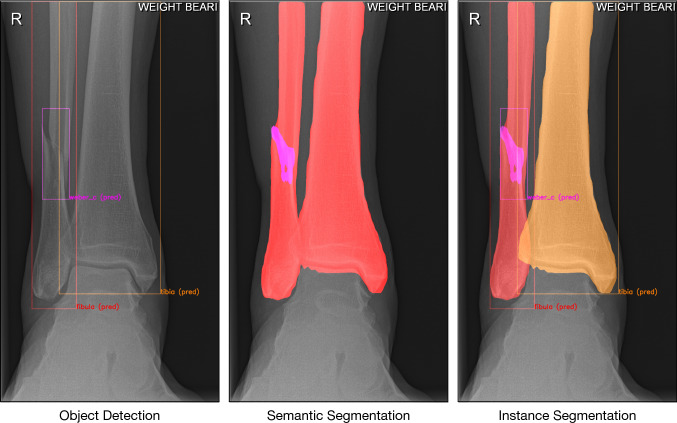
From left to right: Object detection, semantic segmentation, and instance segmentation

An instance segmentation model can segment individual objects (i.e. bones) by combining object detection (bounding box) and semantic segmentation (Fig. 3). The simplified explanation order in which Mask R-CNN does this is as follows (Fig. 2): (1) The radiograph is fed into the CNN; (2) the backbone (ResNet-50) together with the Region Proposal Network (RPN) creates many bounding boxes with each proposal being an object; (3) each region proposal is resized by Region of Interest (RoI) pooling to fit fixed height and width dimensions of 256 × 256; (4a) Mask R-CNN classifies each pixel in a region proposal to create a segmentation; (4b) simultaneously, Mask R-CNN uses object class prediction on each region proposal; (5) predictions are reverted back to original height and width dimensions and projected onto the output image.

### Training of final CNN model

The training set included 326 fracture and 423 non-fracture radiographs, which were labelled and annotated in Labelbox [35]. Standard data augmentation (random cropping and horizontal flip operations) was used to improve the generalization of the model. To reduce bias, cases were re-weighted according to their prevalence. The annotated bounding boxes are used as the Ground Truth for the RPN. Training was completed at 64 epochs (64 iterations of the complete dataset) after 90 min. The training starts from an initial learning rate of 0.05 down to 1/10 every 1000 steps. Each step is commonly known as a mini-batch iteration; in this study, we loaded 12 images per mini-batch.

### Evaluation of final CNN model

Twenty-five patients of each class (AO/OTA 44/Weber A, B, C or No Fracture), were randomly selected by the computer—and cross-checked with the 400 (by the preliminary CNN) selected ‘difficult’ images—for the internal validation set (also known as ‘test set’), to assess the patient-level accuracy. The final prediction was the class with the highest combined prediction value among all radiographic views. The ground truth was the consensus between three experienced fellowship-trained trauma surgeons. Consensus was achieved on all cases; however, ambiguous cases (low inter-observer agreement) were put in a clinically challenging set and swapped with randomly selected patients to ensure objective measurement (high inter-observer agreement) of model performance. After assessing performance using non-ambiguous cases (clinically easy internal validation), these were then put back into the internal validation set to assess the effect of clinically challenging cases (clinically challenging internal validation) on performance of the model.

To assess transportability and generalizability of the model, external validation was performed using 167 cases from our second Level-1 Trauma hospital in the Netherlands with the same methodology as for the internal validation. Due to a difference in protocol, these did not contain mortise views.

**Fig. 4:**
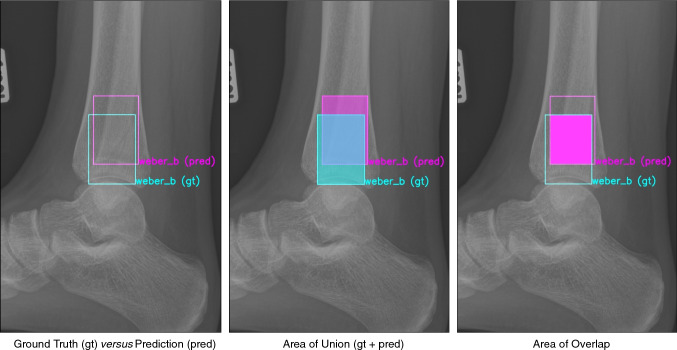
From left to right: Ground truth (gt) versus prediction (pred), area of union (gt + pred), and area of overlap

For assessment of the image-level accuracy, Intersection over Union (IoU; also known as the Jaccard index, Fig. 4) was used to quantify accuracy of the segmentation predictions by the CNN, where 0 indicates no overlap at all and 1 a perfect overlap. Due to the complex nature of CNNs, it cannot be assumed that predictions will perfectly match the ground truth; therefore, IoU is used as it is an indicator of overlap. Generally, an IoU > 0.5 is considered a good prediction [38, 39].

### Statistical analysis

Accuracy (defined as the percentage of cases correctly predicted by the CNN), sensitivity, specificity, and AUC were calculated for each (non) fracture class. The AUC reflects the discriminative ability of the CNN to separate classes, an AUC of 1.0 corresponds to a prediction with perfect discriminatory performance, whereas 0.5 indicates a prediction equal to chance.

Statistical analysis was performed using Python 3.9.0 [Python Software Foundation, Beaverton, United States] with the modules: pandas, cv2, NumPy, sklearn, and plotly.

## Results

### Test set—performance of CNN model 2 on clinically ‘Easy’ cases (Fig. 5)

**Fig. 5:**
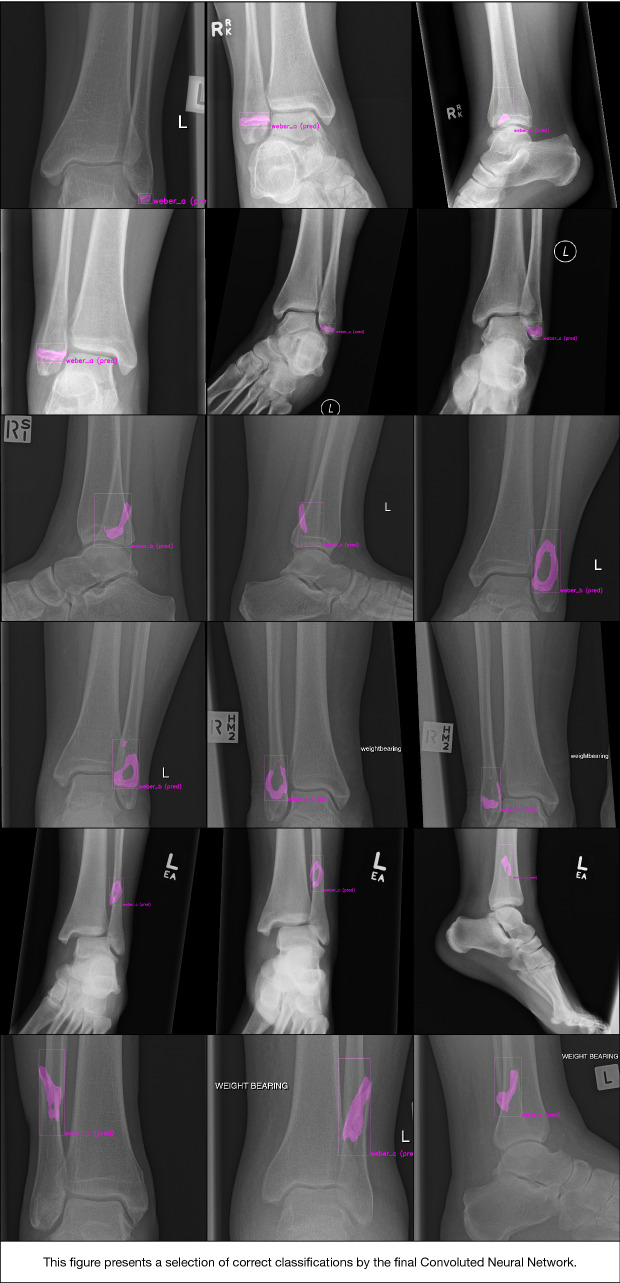
Selection of correct classifications by the final convoluted neural network

The final CNN was able to classify fibula fractures according to four classes (Danis-Weber A, B, C and No Fracture) with AUC values ranging from 0.93–0.99 and 89% accuracy (Tables 1, 2 and Fig. 5). Best accuracy was observed for the ‘No Fracture’ class with 100% and ‘Weber B’ with 92%. Accuracies of 88% and 76% were observed for classes ‘Weber C’ and ‘Weber A’, respectively. Specificity, however, was 100% for both of those two fracture classes.

**Table 1:** Combined radiograph confusion matrix and accuracy

	Predicted				
	Weber A	Weber B	Weber C	No Fracture	Accuracy (%)
Performance on clinically ‘Easy’ cases					
Weber A	19	3	0	3	76
Weber B	0	23	0	2	92
Weber C	0	2	22	1	88
No fracture	0	0	0	25	100
Performance on clinically ‘Difficult’ cases					
Weber A	19	2	0	4	76
Weber B	0	23	0	2	92
Weber C	0	4	18	3	72
No fracture	0	0	0	25	100

**Table 2:** Sensitivity, specificity, and AUC per class

	Sensitivity (%)	Specificity (%)	AUC
Performance on clinically easy cases			
Weber A	76	100	0.93
Weber B	92	93	0.97
Weber C	88	100	0.99
No fracture	100	92	0.99
Performance on clinically difficult cases			
Weber A	76	100	0.93
Weber B	92	93	0.97
Weber C	72	100	0.9
No fracture	100	88	0.98

**Fig. 6:**
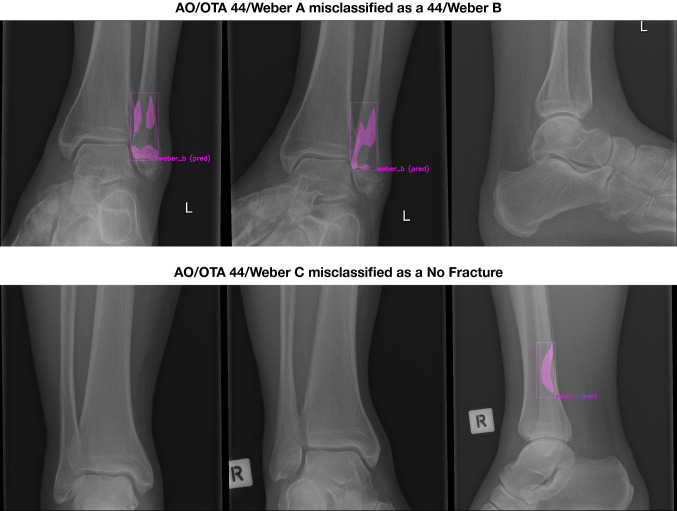
AO/OTA 44/Weber A misclassified as a 44/Weber B, AO/OTA 44/Weber C misclassified as a No Fracture

In the ‘Weber C’ group, three cases were misclassified of which two were subtle fractures that were picked up on the lateral radiograph but missed or misclassified as ‘Weber B’ on the anteroposterior and mortise views, and one was a steep oblique fracture line misclassified as ‘Weber B’. From the ‘Weber A’ group, the six patients that were misclassified, five had transverse fractures at the level of the ankle joint (the line between ‘A’ or ‘B’ classification), and one was a subtle fracture. Two examples of misclassifications are shown in Fig. 6.

### Test set—performance of CNN model 2 on clinically ‘Difficult’ cases

With reintroduction of ambiguous cases, AUC values ranged from 0.90 to 0.98 and accuracy decreased by 4% to an average of 85%. Performance metrics per class are given in Tables 1 and 2. Besides minor changes in other classes, the ‘Weber C’ class was most affected, where accuracy decreased from 88% (22/25) to 72% (18/25). Compared with the initial internal validation set, the ‘Weber C’ class had two extra ‘Weber B’ misclassifications. These occurred with a steep oblique fracture line, and two extra misclassifications as ‘No Fracture’ occurred when there was a presence of high ‘Weber C’ fracture.

### Test set—accuracy of segmentation (i.e. delineation of the fracture line) (Fig. 7)

**Fig. 7:**
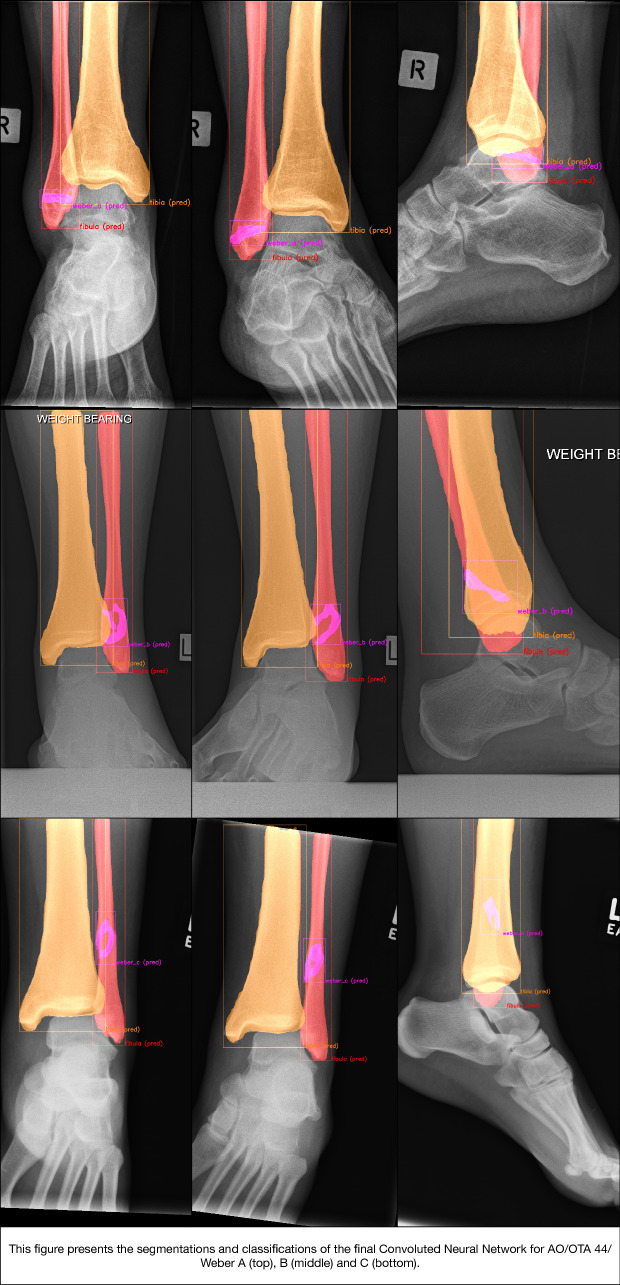
Segmentations and classifications of the final convoluted neural network for AO/OTA 44/Weber A (top), B (middle), and C (bottom)

 Quality of the predicted fracture segmentations by the CNN was quantified by the IoU (Figs. 2 and 7). For the fracture bounding box prediction by the CNN, a mean IoU of 0.65 (SD ± 0.16) was observed. The much more challenging fracture polygon segmentation predictions by the CNN resulted in a mean IoU of 0.47 (SD ± 0.17).

### External validation of CNN model 2

 On the clinically ‘easy’ external validation set without ambiguous cases, the model achieved AUC values ranging from 0.83 to 0.95 and an overall accuracy of 90% (Table 3). Best accuracies of 99% and 92% were achieved for the ‘No Fracture’ and ‘Weber B’ classes, respectively, while the ‘Weber C’ and ‘Weber A’ classes resulted in the least accurate predictions with 71% and 64%, respectively.

**Table 3:** External validation performance

	Accuracy (%)	Sensitivity (%)	Specificity (%)	AUC
Clinically ‘Easy’ external validation				
Weber A	64	64	100	0.88
Weber B	92	92	98	0.98
Weber C	71	71	100	0.83
No fracture	99	99	86	0.9
Clinically ‘Difficult’ external validation				
Weber A	62	62	100	0.86
Weber B	90.0	90.0	96	0.92
Weber C	70.0	70.0	100	0.84
No fracture	99	99	89	0.89

When ambiguous cases were introduced, the model achieved AUC values ranging from 0.84 to 0.92 and accuracy to 89%. Compared to the former external validation, all fracture classes’ accuracies were affected by 1–2% (Table 3). A similar pattern to the internal validation was observed; the model struggled with ambiguous cases; however, in contrast to the internal validation, no specific class was more affected than others.

## Discussion

To date, studies on the clinical application of AI in the field of computer vision have not deployed CNNs to automatically delineate fractures, which can reduce the black box effect as well as aide less experienced doctors who are still in their early learning curve. Moreover, external validity of current CNNs for fracture recognition in orthopaedic trauma is scarce [3]. In this study, we developed a CNN that can detect, classify, and create detailed segmentations of fracture lines in ankle fractures (AO/OTA 44/Weber A, B and C) with an overall accuracy of 89%. In addition, it was found to be externally valid on radiographs from Level I Trauma Centre on a different continent, with an average accuracy of 89–90%. We used a preliminary CNN to select fractures that were difficult to appreciate, aiming for the most efficient training rate per image for the final CNN.

As with any study, this information must be interpreted with respect to its scientific strengths and weaknesses. One limitation is that existing classification systems suffer from varying inter-observer reliability, affecting performance of the model [14]. Another stems from evaluating the CNN using retrospective instead of prospective data, although the internal and external validation were collected from multiple years and thus simulate clinical practice. Also, as training data did not include fractures with concomitant joint dislocations, the CNN is unlikely to recognize this significant fracture subset. These study shortcomings, however, are counterbalanced by several notable merits, including that this is the first paper in the field of orthopaedic trauma to describe a pixel perfect segmentation of fracture lines on plain radiographs—compared to rough predictions using heat/activation maps described in literature—and use a preliminary CNN to select cases to train the final CNN model. Another strength is external validation of the CNN in assessing generalizability and possible bias of the model on data different than that used for development. Moreover, labelling of the internal validation set was done by three independent experienced surgeons, and a consensus was used as the ground truth. Finally, the Mask R-CNN [40] used in this investigation represents a state-of-the-art CNN that accepts entire radiographs as input image for the detection, classification, and segmentation tasks, whereas commonly used CNN models often warrant cropping and are more difficult to implement in clinical practice.

In 2020, Olczak et al. [8] were the first and the only ones thus far to report the use of a CNN in classifying ankle fractures. The current study adds to our knowledge by presenting a CNN that was developed to create a detailed localization and segmentation of fracture lines on radiographs. This may improve clinical reasoning and diagnostics by giving junior clinicians a visual guide and simultaneously reduces the ominous ‘black box’ effect, which facilitates a feedback loop for an ongoing learning curve. Furthermore, this study reports an improvement in the discriminatory performance (AUC values) compared to the study from Olczak and colleagues [8], using less than a fourth (approximately 250 cases) of the 1064 cases for the corresponding classes without tibial involvement (AO/OTA 44A1, B1, B2.1 and C1.1). Accuracy cannot be compared as the latter study did not report an accuracy of their CNN in classifying ankle fractures. As labelling and annotating is very labour-intensive and qualified experts’ time is often limited, training an initial CNN to select optimal cases for training the CNN increases efficiency.

Although accuracy and the AUCs were high, the CNN misclassified 11 out of 100 patients in our test set (Figs. 5, 6, and 7). It should be noted, however, that the AUC is close to 1 (indicating almost perfect discriminatory performance), ranging from 0.93 to 0.99. Accuracy depends greatly on individual cases in the internal validation set. Interestingly, the same cases considered ambiguous by surgeons (i.e. poor inter-observer reliability) were also the ones that CNN had difficulties with. Since CNNs can only be as good as their training, it should be noted that without an absolute truth (e.g. a CT scan), current computers can only be trained to approach the performance of surgeons—but they cannot surpass it. If one defines the ground truth as a consensus agreement, however, at least some inherent surgeon bias can be eliminated. The AO/OTA 44/Weber A and C classifications were most susceptible for misclassification, together accounting for nine of the 11 errors. The recognition of higher Weber C-type injuries might be limited by that the fact that the CNN appears to have no positional awareness and seems to classify based purely on fracture configuration. Another explanation might be the alternative CNN shortcoming in trying to detect features that exist at the margin of an image; similarly, CNNs are likely dependent on the variability in what gets captured in a given radiograph, since for multiple reasons this clearly varies image to image. Since Weber C injuries can have dynamic instability or exist at a level not identified on non-stressed or more limited exposure radiographs, it makes sense that this is where these algorithms seems to fall short and demonstrate room for improvement. For the segmentation task, the average IoU value for the bounding boxes was good, even though one study suggests that IoU is optimal for round shapes, but not for elongated ones [41] such as those used in this study. As expected, the highly variable fracture line segmentations resulted in a lower IoU compared to the bounding box. However, the average IoU was still close to 0.5, suggesting an overall accurate fracture line segmentation despite the great variation in fracture configurations.

It is worth noting that accuracy was the highest when detecting a lack of fracture, doing so with 100% accuracy. Thus, while much of the efforts of this study were to distinguish between various fracture patterns, the ability of the same process to exclude fracture is inherently useful to those working in the emergency or urgent care setting who simply need guidance as to when to seek additional orthopaedic consultation.

Performance is often reduced when assessed with an external validation set [4, 16, 42], as there are many observer- and machine-dependent variances between hospitals. Therefore, geographical external validation is a stringent and crucial test towards clinical implementation of these models. Enabling the external validation set to usually have two views (anteroposterior and lateral) instead of three (mortise) improved classification of fractures that were only detected on one of the views, with a caveat that the CNN has to be more confident of its ‘Fracture’ classification than its ‘No Fracture’ classification. A notable distinction here is that when there were three views and the fracture was seen on the lateral view but not on the anteroposterior and mortise, it was always classified as ‘No Fracture’.

In summary, this early work on automated detection in orthopaedic imaging demonstrates remarkable future potential despite several shortcomings noted in its current level of development. In conclusion, even though object detection has been employed for certain other types of fractures and imaging modalities, this study presents the first automated segmentation of fracture lines on ankle radiographs. The accuracy and AUC values presented in this paper certainly fortify a role for CNNs in detecting and classifying ankle fractures. Moreover, using a preliminary CNN to identify cases resulted in a network that was accurate enough to be externally valid in another hospital, surely important for reducing the workload of creating high-quality data for training of CNNs.
